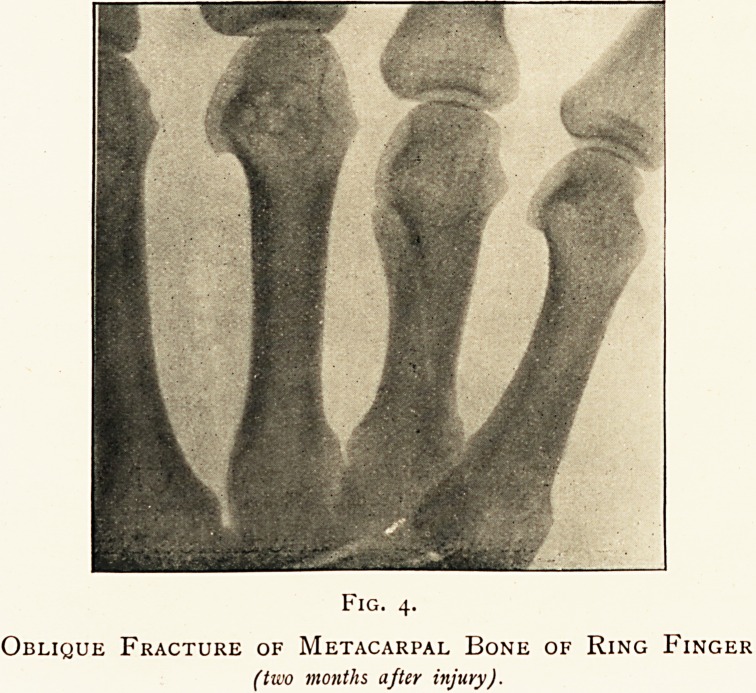# A Simple Form of Influence Machine for X-Ray Work

**Published:** 1899-09

**Authors:** William Cotton


### A SIMPLE FORM OF INFLUENCE MACHINE FOR X-RAY WORK.

BY

William Cotton, M.A., M.D. Ed.

To get X-rays out of a suitable vacuum tube, it needs to be excited by an electric discharge at a tension of hundreds of thousands of volts. Such a discharge is furnished by the induction coil and by the static induction or "influence" machine, associated in its most elegant and effective form with the name of Mr. Wimshurst. In spite of the obvious advantages of the latter type of machine over the former, very little systematic X-ray work with the influence machine has appeared; and the greater number of the text-books of radiography pass the matter over with a general statement that the

Holtz or the Wimshurst machine from the steadiness and continuity of its discharge is specially fitted for work with the screen. It is possible, however, with a very simple form of influence machine to get good X-ray negatives. Such an apparatus is represented in Fig. 1.

FIG. 1.

The essential part consists of two parallel circular discs of ordinary window glass, 22 inches in diameter, about $\frac{1}{8}$ of an inch thick, and $\frac{1}{4}$ of an inch apart, varnished with shellac. Being mounted centrally on independent wooden bosses, they can be rotated in opposite directions on a horizontal metallic spindle or axle, which pierces them and is supported at each end by a wooden upright springing vertically from a stout rectangular mahogany base. The lower parts of the two uprights support another horizontal axle, parallel to the main one, turned by a handle, with two wooden wheels on it, which by a crossed and an open strap act respectively on the bosses of the glass discs and thus supply the necessary motive power. The glass employed is of the gauge of 22 ounces per square foot. It must be of the highest dielectric quality; green glass and plate glass are found unsuitable, and of white glass of similar commercial standard some specimens are found on trial much fitter than others. Ebonite discs do very well, but exposure to vicissitudes of temperature causes them to bend and buckle, and in time the atmosphere affects them chemically on the surface and so deteriorates their insulating property.

At each end of the main axle where it projects through the supporting upright is metallically connected a movable brass rod, known as the neutralising rod. This rod is bent round at each end towards the surface of the adjacent glass disc, and has jointed to it at each end a straight piece about four inches long, radially disposed as regards the glass disc, parallel to its surface and just within the outer margin thereof. Each straight piece has attached to it at equal distances so as lightly to trail upon the surface of the glass three fine wire brushes. Thus we have two neutralising rods, a near one and a far one, each with two straight pieces, and each straight piece has three brushes,

*i.e.*, there are twelve neutralising brushes in all. In Fig. 1 the greater part of the neutralising rod on the near side of the discs is seen, with its upper straight piece; the upper straight piece and brushes of the farther neutralising rod are dimly seen through the two glass discs.

Besides the wooden uprights, there are situated upon the rectangular base of the apparatus and towards its extremities three other pairs of structures. At the inside or far side of the base are two insulating cups or holders for a pair of Leyden jars, should it be thought advisable to join them up in the circuit. In front of each holder and opposite the right and left lateral rims of the glass discs are two stout vertical rods of ebonite or other insulating material, supporting a polished brass sphere about three inches in diameter—the prime conductors. From the metallic neck of each prime conductor there runs in horizontally for about four inches and exactly in the line of the horizontal diameter of the glass discs a pair of brass collecting rods—one rod in front of the near disc, the other behind the farther one. Each collecting rod has on it at equal distances eight trailing brushes similar to those on the neutralising rods. Thus we have two prime conductors—a right one and a left one—each with two collecting rods, and each collecting rod has eight brushes, *i.e.*, there are thirty-two collecting brushes in all. In front of the insulating supports of the prime conductors is a pair of more slender insulating rods to support the metallic stems of two approximately hemispherical knobs, somewhat smaller than the spherical heads of the prime conductors, which may be called the secondary conductors. The metallic stem is bent to a right angle. The supporting insulating rods can be rotated round the vertical axis and fixed at any point by a small set screw, so that the gap—the spark gap—between the prime and secondary conductor of each side is an adjustable one. It is on the management of these spark gaps, in series with the vacuum tube, that success or failure in the emission of X-rays from the tube mainly depends in the case of the influence machine.

The remainder of the apparatus as ordinarily joined up consists of two well-insulated thick copper wires or "leads," coiled *secundum artem*, running over from the brass stem of each secondary conductor to the external wire terminal of the indispensable Jackson focus tube on the same side. The stem of the tube is held by a specially designed well-insulated stand, with well-insulated ebonite rods to support the wire leads and to take the strain of them from off the external wire terminals of the tube. It is useful in some cases to have some arrangement for a second spark gap in series on each side of the tube; and it is always of importance to have the leads connected with the respective external wire terminals of the tube under cover of small hollow tunnelled brass spheres to minimise leakage of electricity from sharp points by brush discharge outside the tube. The internal resistance of the focus tube best suited for this machine is so great, that the distance apart of the external wire terminals of the electrodes into the tube should be greater than the spark length of the machine in air with tube away, with the same view of preventing brush discharge. To avoid the risk of puncture of the tube by sparking, the tube should have its external wire terminals at least eight or ten inches apart.

With the machine described a pair of Leyden jars may be used in two ways. *First:* The secondary conductors being disconnected from the tube and connected one to another by a thick copper wire between the brass stems, the spark gap between prime and secondary conductor on each side is left open, and the inside of each Leyden jar is put in direct communication with the adjacent conductor. Each jar is on its insulating cup, and has its outside coating connected by a thick copper lead to an external terminal of the tube. When the accumulated unlike internal charges of the Leyden jars recombine across the spark gaps and the wire uniting the secondary conductors, we have the reverse charges in the outer coatings recombining across the tube. As a result we have one or two explosive discharges per second, with deafening noise and an almost blinding yellow fluorescence of the tube; the screen lights up most brilliantly, but only for a moment. Owing probably to the higher quantity (amperage) of the electrical discharge, this method is dangerous to operator and patient, and very detrimental to the tube: it is only to be used with great caution in radiographing the thickest parts of the body. *Second:* When the apparatus is joined up in the ordinary way, a well-insulated Leyden jar (with the knob from its interior in contact with the adjacent prime conductor), at each side, having its outer coating removed, promises to be of great use. When this arrangement is made, the discharge across the spark gaps is white instead of violet, and is continuous. In this case the jars act as condensers or reservoirs of electricity.

The machine described, like other influence machines which have no metallic sectors, plates, buttons, or knobs upon the discs, is not (except under the most unusual climatic conditions) self-exciting—in other words, the discs are revolved with no electrical effect unless an initial charge of + or − electricity is communicated to one or other of the prime conductors. Once excited, it will not require re-excitement for a whole evening though revolved very slowly, or even allowed to be at rest for a little while. The most convenient method is to have a miniature influence machine, called the "Exciter," of the type which is the best known of Mr. Wimshurst's numerous modifications of the influence machine (shown in Fig. 2, in position to give a − charge of

FIG. 2.

electricity to the left-hand conductor of its big relation). The sectors render it self-exciting—it has two glass discs seven inches in diameter, each with twenty-four tinfoil sectors on outside face, one brush on each neutralising rod to touch the passing sectors, and brushes on the collecting rods. There is a $1\frac{1}{2}$-inch spark. For exciting purposes, only one prime conductor is needed on it; the other is superfluous,

It will at once be seen that structurally the two machines are almost identical. The influence machine described at length is a sectorless Wimshurst—it is a Wimshurst without sectors, with trailing brushes at the collecting and neutralising rods instead of points that are not in contact and brushes that merely touch the metallic sectors. In practice the sectorless Wimshurst is to be preferred to a sectored one of the same size for five reasons :—

(1) It is easier to construct and keep in order—it is simpler.

(2) It never reverses polarity during running—the other does so occasionally.

(3) It has a greater output of electrical energy—the tube lights up better with the same amount of mechanical energy expended on the handle.

(4) In a sectored Wimshurst, where there is a great resistance in the tube, the inner ends of the sectors leak towards the boss, as shown by a visible brush discharge.

(5) After prolonged use the friction of the brushes on the sectors as they pass leads to the formation of rings of metallic deposit, which break down the insulation.

On the other hand, except under exceptional circumstances, the sectorless Wimshurst is not, and the sectored Wimshurst is, self-exciting.

The sectorless Wimshurst having been carefully dusted, has a charge communicated by spark or contact from the exciter, and the discs are revolved. One soon learns which is the proper prime conductor to charge, according as the cathode or anode is left or right in circuit. Most operators find it convenient to turn the handle from left to right over the top of the circle. The energy to be expended is quite within the ability of an intelligent small boy. It is not a very high form of skilled labour to turn a handle steadily, and as a rule there is no lack of willing amateur assistance available, so that the medical man's attention can be entirely devoted to keeping the injured part and the patient steady. The most effective output of electricity is got when the winch is rotated eighty times a minute, giving 200 revolutions in the same time to each glass disc in opposite directions. The spark gaps are opened gradually from contact till the tube fluoresces brilliantly, which it never fails to do; unless there is actually damp deposited on the outside of the tube, the spirit lamp is entirely unnecessary. The focus tube to be preferred is one adapted for a nominally 10-inch spark induction coil: the sectorless Wimshurst gives with Leyden jars a maximum spark of 10½ inches; without, in favourable conditions of atmospheric dryness, a continuous brush discharge of 9½ inches in length between terminals when the tube is removed. All the brass work of the conductors should be of the highest finish, to avoid the formation of any pointed parts and consequent brush discharge owing to local increase of electric density; and for the same reason care must be taken that if any silk fastenings are made at the external terminals of the tube the knotted ends be cut short. Otherwise we shall get a large part of the discharge streaming away visibly. Bedclothes in the neighbourhood of the circuit should be put out of the way, for if they are heaped up towards the tube, the moisture in them streams up and locally breaks down the atmospheric insulation. The apparatus, joined up in the ordinary way, is free from noisiness—there is only a low-pitched hissing sound. The odour associated with the presence of ozone is very perceptible. It is found that the best results are obtained when the upper end of the nearer neutralising rod is situated 30° to the left of the vertical, and that of the farther one the same distance to the right (the "five minutes to five" position).

The self-excitement of the sectored Wimshurst on rotation is commonly explained by saying that when there are a number of metallic insulated plates scattered over a large non-conducting surface, one or other of them is pretty sure to be above or below the rest in electric potential. In other words, there is always an initial charge somewhere about. If the *rationale* of action of the ancestral electrophorus of Volta be kept in mind, it is comparatively easy in a general way to understand how by mutual induction (of each on all the rest) the metallic sectors of a sectored Wimshurst, one or other of which has a charge in it, can on rotation give rise to a continuous heaping up of + electricity at one prime conductor and of − electricity at the other, while the neutralising rods by the touch of their brushes on the passing sectors play the part of the touching finger on the "shield" of the electrophorus. The sectorless Wimshurst, once it has an initial charge given to one of its prime conductors, produces, in a manner that is very obscure, a similar heaping up of a + and a − electricity at opposite conductors. Mr. Wimshurst suggests that in the sectorless machine the trailing brushes (which ones?) act as sectors. Finally, the recombining electricities discharge across the vacuum tube put in the path of their reunion.

The results obtained by means of the sectorless Wimshurst described may be given under two heads.

I.—**With the Screen.**

I was able to see the shadow of a watch through the trunk of a thickset man; the heart with the pericardial bag was quite distinctly seen in outline suspended in the thorax of a boy of ten viewed from the back; in a young lady of twelve, the waves of contraction of the heart muscle on its left border were seen quite deliberately passing downwards in regular succession, viewed from the front of the chest; it was always possible with the Leyden jars joined up to get the fluorescent glow on the screen through the adult trunk. Every detail of the bones, even the fissure of a fracture if caught in its plane, could be seen in the hand, wrist, elbow, and ankle of the adult, and the shoulder and thorax of a young boy. In all these parts we get indications of soft tissues. The bones of the phalanges and metacarpus could be distinguished five feet from the tube through a wooden door; and at the same distance the screen lit up through a brick partition.

It is an interesting point to note that the fluorescent screen glows with a light that has very little actinic power. Photographs with an ordinary camera of objects shadowed on the screen have been taken successfully, but I have never seen any, and have failed again and again to obtain a negative at different distances and with different kinds of plates, even after an exposure of ten minutes. On the other hand, it is easy to obtain a good photograph of a fluorescing focus tube with an exposure of a quarter of a minute; while everyone knows the electric spark under the same conditions photographs itself instantaneously.

Fig. 3.

Oblique Fracture lower $\frac{1}{3}$ of Tibia in Adult.

Fig. 4.

Oblique Fracture of Metacarpal Bone of Ring Finger

*(two months after injury).*

To employ a screen to the best advantage the room must be darkened, and the eyes prepared by being kept in a dimly lighted room for ten or fifteen minutes. Daylight should always be religiously excluded.

II.—**With the Photographic Plate.**

The duration of exposure is somewhat long; about half the time would be sufficient where the detection of the presence only of a metallic foreign body is needed. We get pictures with detail and sharpness and quite a perspective effect, not mere shadowgraphs. To do justice to the negative, I always use gelatino-chloride paper, slightly undertone, and squeegee on polished glass. Shortness of exposure for its own sake is not much of an object when using the Wimshurst apparatus, as the patient keeps placid, and the operator is not obsessed by the haunting idea of impending collapse to one or more parts of a long train of complicated and costly apparatus. To give examples of the degree of detail, the sesamoid bones of the hand and foot (including that in the tendon of the peroneus longus as it crosses the sole of the foot) came out sharply, and we get details of all the bones of wrist, elbow, ankle, and knee, especially in the adult—the fibrous structure of the os calcis and astragalus is quite distinctly shown. Time of exposure: *Adult:* Fingers, 2 minutes; hand, 8—10 minutes; forearm, 10 minutes; elbow, 10 minutes; shaft of humerus, 10 minutes; shoulder (with clavicle, ribs, and scapula), 45 minutes; foot and ankle, 16—20 minutes; knee and leg, 20 minutes. *Aged 12:* Hip-joint, showing pelvic brim, 20 minutes. *Aged 6:* Femur, shaft, 18—20 minutes. *Fœtus, 6—7 months:* 15 minutes.

Among my own cases were fracture of upper part of ulna in a boy of 10; fracture of the shaft of the femur in a boy of 6 on the 24th day; oblique fracture of the lower end of tibia in an adult (see Fig. 3, where the two ankles above and below to the left were taken on the 29th day, the others on the 96th): fractures of the metacarpal bones of the thumb in adult and of the ring finger in adult on the 4th and 64th days (for this last see Fig. 4); in these and in others the fragments are seen in perspective and, as it were, one through another where they overlap. Those figured have been chosen to show the easily available range of the apparatus described. In two cases of suspected renal calculus, after two trials, in each case of about 35—45 minutes each, no stone appeared in the negative, only the indication of some ribs in the thinner (female) subject, and of the iliac crest in the other (a male). In both cases, at both trials, sufficient rays were got through to bring out the shadow of a coin lying on the envelope of the photographic plate. In the first case I was quite satisfied that if an oxalate of lime calculus—which is the most opaque of calculi to X-rays—had been present, it would have appeared on the negative. To be successful in these cases a more powerful apparatus is needed, say one with two pairs of discs 22 inches in diameter, or with two discs 36 inches in diameter; or possibly the same apparatus would do with a very soft tube.

In the cases enumerated, the average distance of the platinum plate from the photographic plate would be about 12 inches. At this distance a whole plate uses up only about $\frac{1}{15}$ of the available area of X-rays, and at 15 inches only about $\frac{1}{24}$, assuming the rays to radiate with equal intensity from the anti-cathodal centre of emission over just a little less than a hemisphere.[1] Some day we shall learn how to economise our X-rays a little better.

Dr. Monell[2] states that his apparatus (constructed originally for therapeutic purposes) consisted of an 8-plate 30-inch Holtz machine, not self-exciting, driven by a $\frac{1}{6}$-horse-power motor. He took a detailed radiogram (14 by 17 inches) of a fully clothed adult woman from a distance of twenty-five inches in five minutes. He undertakes to get negatives of the hip-joint, of renal calculus, or metallic bodies anywhere in the trunk of the adult in five minutes at twelve or fifteen inches distance. He publishes no radiogram, nor have I been able in any of the text-books, including those mentioned at the end of this paper, to find any radiogram avowedly obtained by means of an influence machine, except one[3] of a purse containing a key and

1 "Further Observations on the Properties of X Rays," by W. C. Roentgen, translated in *Arch. Roentgen Ray*, 1898-99, iii. 80, 82.

2 *Manual of Static Electricity in X-Ray and Therapeutic Uses*, 1897.

3 *Roentgen Rays and the Phenomena of the Anode and Cathode*, E. P. Thompson, 1896, p. 99.

two coins obtained in sixty minutes, and another[1] of a bird showing excellent detail, and one[2] of a frog, of which the same may be said.

The ordinary process blocks of the periodicals are very unsatisfactory in reproducing the delicate gradation and detail of Wimshurst negatives. Lantern slides by reduction from the negative are also unsatisfactory. Lantern slides by contact are better; and lantern slides by reduction from a glazed silver print on gelatino-chloride paper are best of all. In this case, after taking precautions to avoid flares from the highly-glazed surface of the print, a quarter-plate process plate negative is taken in an ordinary camera; and the negative when developed for contrast is intensified slightly for the finer detail. Lantern slides are then taken from the process negative by contact, or prints may be got in miniature of the original ones. The method is further useful when we wish to show by lantern on the sheet a grouping together of different prints. After all, nothing comes up to the original highly finished gelatino-chloride positives. In every case the photographer must have the usual methods of negative development and intensification at his finger-ends to get the best results of his X-ray exposures.

The advantages of an influence machine in X-ray work may now be considered, taking as a standard of comparison an eight- or ten-inch spark induction coil, with special reference to the sectorless Wimshurst described.

(1) The primary inducing current of the coil and its appurtenances are abolished. There is no primary battery or accumulator, no interrupter, no primary coil, and consequently none of the uncertainties, annoyances, or risks of breakdown associated with the use of these pieces of mechanism. Further, the absence of Fizeau's condenser (a necessary part of the primary circuit to render the discharge of the secondary coil unipolar and to minimise the sparking at the platinum contacts of the interrupter) removes one source of "fault" development (and breakdown by sparking penetration of an insu-

1 *The A.B.C. of the X-rays*, W. H. Meadowcroft.

2 *Arch. Roentgen Ray*, 1898-99, iii. pl. LV.

lating partition) somewhere over its forty or fifty square feet of area—a fault that is difficult to locate, reach, or repair. A similar remark applies to the secondary coil of the induction coil proper, with its six or eight miles of fine insulated wire, which at any point, from overheating or mechanical injury or sparking perforation due to overdriving, may likewise develop a "fault." In place are substituted two glass discs in the open, easily removed and replaced if broken.

(2) Owing to the small amount of electricity, though at a high tension, passing continuously and regularly, the life of the tube as regards useful work is a prolonged one. There is none of the heating of the internal terminals of the tube under the heavy, interrupted, and it may be irregular discharges of the induction coil. The tube, not a very soft one to begin with, used for the last year with the sectorless Wimshurst has been under continuous discharge for a total of over 160 hours, and has only recently shown signs of "hardening." This hardening over its first condition is probably due to the use of the Leyden jars, for a few hours in the aggregate, by means of their outer coatings, in the manner described. When joined up in the ordinary way the tube never appears to heat and has never failed to respond promptly to the excitation of the discharge. The only difference to be noted is that the spark gaps have to be a little wider than they were a year ago. The tube has never turned sulky or needed to be coaxed with the spirit lamp or by any other of the empirical methods resorted to by the practical worker with the coil. It is probable that with the coil no tube after the first dozen hours of exposure to actual discharge but is perceptibly hardened, especially if the capacity of the apparatus is forced to its utmost. The sharpness of the Wimshurst negative is probably due in part to the quiet continuous discharge, the small emitting area not shifting, nor the original fine adjustment of the cathode and anticathode altered by irregular or extreme heatings under greatly varying discharges. To put the matter in a nutshell, it is hardly to be expected that any induction coil set, worked at a strain to perform some *tour de force*, however successful at the time, will repeat the performance, while a static induction machine may be almost surely relied on to do again what it has once done.

(3) It is generally acknowledged that the continuous non-flickering, uniform discharge, varying only with the rate of rotation of the discs up to a maximum, of the influence machine renders it more suitable than the induction coil for work with the fluorescent screen. But, further, it is peculiarly fitted, especially if driven by a well-regulated electric or other motor, for delicate and prolonged experimentation, *e.g.* for photometric observation of the intensity of the fluorescence under different conditions ; for examining into the influence of X-rays on bacteria in the animal body or in cultures ; for testing the action of the rays on growing plants or germinating seeds. All such experiments when made with a coil labour under the fallacy that nothing certain is known quantitatively about the agent employed, except that it is on the whole after a short time irregularly diminishing in energy. The Wimshurst apparatus is the only one at present known in X-ray work that is adapted for the standardising of X-ray electrical, fluorescent and other physico-chemical effects.

(4) When joined up in the ordinary way without Leyden jars the static induction machine gives a discharge which is absolutely harmless to operator or patient, even if the live wires be purposely handled. A pricking sensation hardly amounting to pain and a fading of fluorescence in the tube is the sole result. There are none of the dangerous shocks to be apprehended from accidentally touching the circuit of the induction coil, on account of the small amperage associated with the continuous discharge of the former apparatus. No cases of eczema have been reported with the influence machine as yet; it would be very interesting to test the matter by exposing to X-rays from a tube excited by an influence machine individuals who are known to be susceptible to X-rays and to have previously suffered from exposure to a tube excited by a coil. In demonstrating his fluorescent screen, the demonstrator has had to be exposed to the rays of the tube with his trunk in close neighbourhood thereto for four and a half hours without any inconvenience.

(5) In regard to portability, an important question as regards cases that have to be X-rayed in their own bedrooms, it may be mentioned that the sectorless Wimshurst weighs 28 lbs., the Leyden jars 3 lbs. each, the focus tube and stand 7 lbs., and the exciter 7 lbs.—in all 45 lbs.,—just a little over the weight of a six-celled charged lithanode accumulator; the jars are not indispensable, and in this case the apparatus weighs rather less. The whole apparatus can be packed in a box 39 × 19 × 14 inches, which can be used as a pedestal.

(6) The cost of an apparatus is about half or a third of a coil set giving the same length of spark; and there is no cost for maintenance or repairs, there being no accumulators to re-charge or parts to wear out.

The objection most urged against the influence machine is that it is liable to be much affected by damp and dust. Most certainly it is not more so than other electrical apparatus, and with ordinary care there is no great difficulty in keeping it in working order. Mr. Wimshurst says his machines work in the damp; he keeps them in glass cases permanently, to avoid dust and handling by the inexperienced. The sectorless Holtz is more susceptible to damp, and requires to be kept permanently in a case with a bowl of chloride of calcium, changed every six months, to keep the air dry. The sectorless Wimshurst described is kept in an ordinary room without any casing, and taken without packing in a cab to the patient's house; and if the night be damp, a little warming at a fire and dusting with a silk handkerchief is all that is required. On only one occasion I have seen it make a failure, and then it was on a very damp night in a long damp vault of a schoolroom, set up for use on a platform that had just been brought in from lying in the drizzle. Even under these circumstances we got a fair negative of metallic objects, and could see the bones of a man's fingers on the screen.

This paper has been written in collaboration with Mr. Thomas Clark, of Horfield, Bristol. He is almost entirely responsible for the electrical part of it. Some time ago he experimented successfully for X-rays with a sectored Wimshurst of his own construction, and was led to devise and construct the sectorless modification described above. His apparatus and some of the resulting radiograms were demonstrated lately by means of photographs and lantern slides before the Roentgen Society of London, and roused considerable interest among the electricians and medical men present. Having worked previously with coil and accumulator, I can speak from personal experience of the advantages and convenience of his system, especially in patients' own homes.

The Roentgen ray is the gift of Experimental Science to the Art of Medicine; it remains to be seen what uses the physician will make of this munificent present from the physicist. Simplicity of apparatus, provided we have efficiency and reliability for ordinary purposes, is the necessary condition for a general application of the new light to diagnosis by the practitioner. It is interesting to note that the evolution of the existing therapeutic influence machines for X-ray purposes has been in the direction of simplification, whereas the induction coil tends every day to become increasingly elaborate, complicated, and costly.

Mr. Wimshurst's latest expressed opinion is that "in a few years influence machines will be the only generators of electricity in use for X-ray purposes."

### BIBLIOGRAPHY.

*References marked * I have been unable to verify myself.*

*A*. As regards the **history and theory** of the **influence machine,** a good deal of information is given in standard electrical textbooks. (The trade catalogues which electrical manufacturing firms give are interesting as regards the form of apparatus, and different forms of Wimshurst are described and figured in *Engineering*, vol. xxxiv., p. 323, May 21, 1886; vol. xxxv., p. 4, April 17, 1891; vol. xxxix., p. 60, May 5, 1893; vol. xxxix., p. 490, May 21, 1897; Oct. 6, 1882: and the *English Mechanic*, Nov. 14, 1884; Jan. 16, 1885; May 1, 1885; Oct. 10, 1885; Nov. 6, 1885.)

"Influence Electrical Machines," a lecture delivered by Mr. James Wimshurst to the Royal Institution, April 27, 1888.

"Influence Machines from 1788 to 1888," Prof. Silvanus Thompson: an address to the Society of Telegraph Engineers and Electricians, with discussion, *The Electrician*, June 8, 15, 22 and 29, 1888.

*Electric Influence Machines*, by J. Gray, 1890.

"Wimshurst Alternating Influence Machine," *English Mechanic*, Nov. 27, 1891.

"Alternating and Experimental Influence Machine," Mr. James Wimshurst, *Proceedings of the Physical Society of London*, Dec. 1871, and *Philosophical Magazine*, June, 1891.

"A New Form of Influence Machine," by James Wimshurst; and

"An Influence Machine," by W. R. Pidgeon, *Proceedings of the Physical Society of London*, Dec., 1873.

*B.* As regards the **use** of the **influence machine** for **X-rays**.

*Practical Radiography*, by A. W. Isenthal and H. Snowden Ward, 1896, gives some practical details of early work with the Wimshurst machine.

* "Radiography," by W. Wilson, *Photography*, vol. viii., p. 708.

* "Experiments on Röntgen Rays," by T. C. Porter, *Nature*, 1896, liv. 149; 1896–97, lv. 30.

* "Production of X-Rays," by Prof. Oliver Lodge, *Nature*, 1896-97, lv. 100.

*Manual of Static Electricity in X-ray and Therapeutic Uses*, by S. H. Monell, 1897.

*Radiography*, by G. R. Bottone, chaps. vi. and vii., 1898.

"The Advantages of the Influence Machine for Lighting X Ray Tubes," by Mr. James Wimshurst; paper read to the Roentgen Society, *Arch. Roentgen Ray*, 1898, ii. 83.

"The Influence Machine in X Ray Work," by Heber Robarts, M.D., abstracted in *Arch. Roentgen Ray*, 1898–99, iii. 104.

## Figures and Tables

**Fig. 1. f1:**
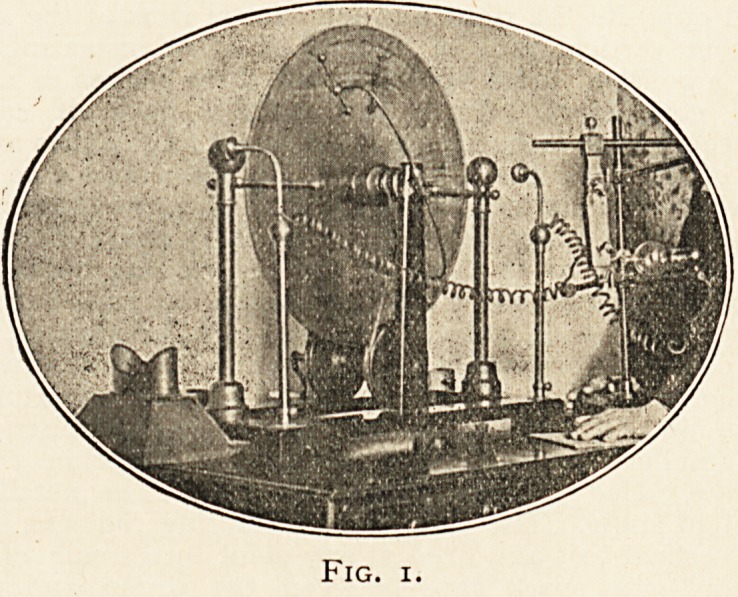


**Fig. 2. f2:**
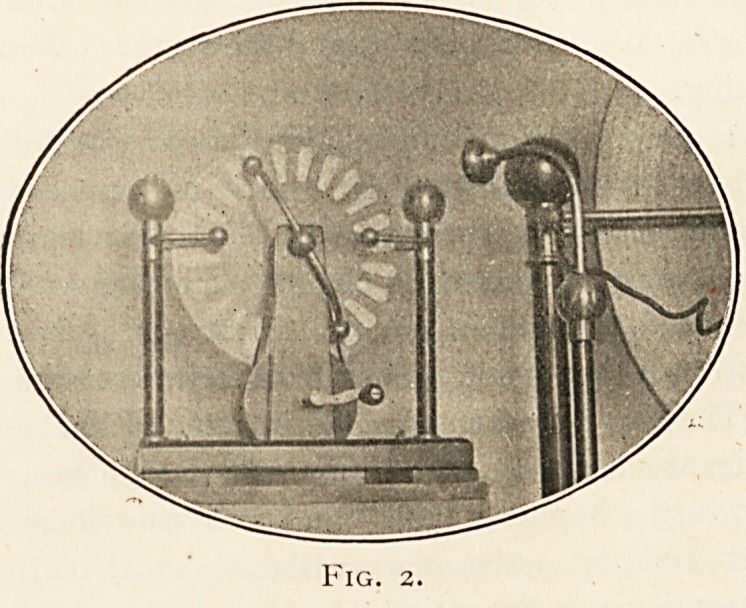


**Fig. 3. f3:**
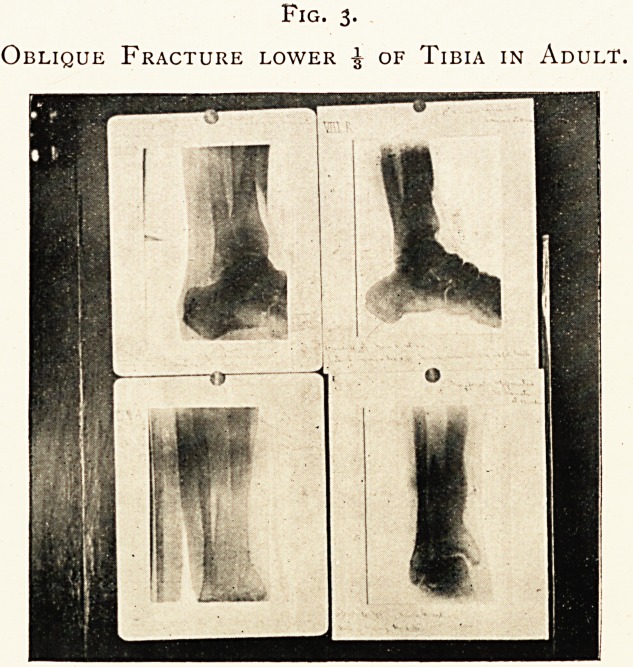


**Fig. 4. f4:**